# Blockchain research in healthcare: a bibliometric review and current research trends

**DOI:** 10.1007/s42488-021-00046-2

**Published:** 2021-04-04

**Authors:** Abderahman Rejeb, Horst Treiblmaier, Karim Rejeb, Suhaiza Zailani

**Affiliations:** 1 Doctoral School of Regional Sciences and Business Administration, Széchenyi István University, Győr, 9026 Hungary; 2 Modul University Vienna, Am Kahlenberg 1, 1190 Vienna, Austria; 3 Higher Institute of Computer Science El Manar, 2, Rue Abou Raïhan El Bayrouni, 2080 Ariana, Tunisia; 4 Faculty of Business and Accountancy, University of Malaya, Kuala Lumpur, Malaysia

**Keywords:** Blockchain, Healthcare, Review, Bibliometrics

## Abstract

The literature on blockchain-enabled use cases has grown exponentially over recent years. Yet, studies are missing that apply bibliometrics and visualization techniques to unravel the dynamics and current discussions pertaining to the nexus of blockchain technology (BCT) and the healthcare field. To close this knowledge gap, we examine the knowledge base and research hotspots of BCT research in the field of healthcare. We carry out a series of bibliometric analyses on the extant literature, including the scholarly production, developmental pattern of the annual total number of authors, and identification of productive academic institutions, countries, and leading authors. Additionally, we conduct a keyword co-occurrence analysis and identify the major research hotspots and trends for the future. The findings of this research are valuable for scholars and practitioners who seek to better understand the development status, dynamics, and trends pertaining to BCT in healthcare.

## Introduction

Along with the major advances that have been achieved in the healthcare field recently, the emergence of blockchain technology has led to several proposed solutions regarding the shortcomings of public and private health information technology systems (Randall et al., 2017; Stafford & Treiblmaier, 2020). BCT was popularized by Satoshi Nakamoto, a mysterious person or group who designed Bitcoin, the world’s first cryptocurrency (Nakamoto, 2008). In the white paper entitled “Bitcoin: A peer-to-peer electronic cash system,” Nakamoto (Nakamoto, 2008) proposed the concept of creating a cryptographically-secured and decentralized currency that facilitates financial transactions. The core technological innovation of the Bitcoin protocol is its ability to solve the double-spending problem and to transfer money electronically without the use of a central third party (e.g., banks) that is needed to authorize transactions (Hanley, 2018). According to Treiblmaier (Treiblmaier, 2018), blockchain (BC) can be defined as a “*digital, decentralized, and distributed ledger in which transactions are logged and added in chronological order with the goal of creating permanent and tamperproof records*” (p. 574). BC is not a single technology, but rather a combination of multiple technologies, tools, and methods that are leveraged to address numerous business use cases (Rejeb et al., 2018; Treiblmaier, 2019; Rejeb et al., 2020).

In recent years, the healthcare industry has identified BC as a flexible technology with far-reaching potential. In this regard, Wang et al. (Wang et al., 2018) claim that organizations embarking on BCT would be able to guarantee fast healthcare interoperability, user-oriented medical research, and counterfeit drug prevention and detection. BCT can help to increase the accuracy of health diagnoses in cases where security and privacy pose additional challenges for the healthcare system (Zhang & Lin, 2018). The intersection of healthcare and BCT has encouraged the medical community to embrace new practices that change the entire structure of the healthcare ecosystem. For example, IBM Watson engaged in a two-year agreement with the US Federal Drug Agency to implement BCT in order to secure the sharing of patient data (Carpio,  2018). Another example is the US Center for Disease Control and Prevention, which tests various capabilities of the technology, including time stamping, peer-to-peer reporting, and processing functionalities to identify disease outbreaks in real time (Alkhaldi, 2020). Apart from the US, several other countries are currently evaluating the implementation of BCT in the healthcare sector, including China (Wang et al., 2018) and Switzerland (Zhou et al., 2019).

Blockchain (BC) research has recently received extensive attention from healthcare academicians and practitioners for several reasons. The introduction of BCT has provided workable solutions for overcoming the key issues that have plagued the healthcare system for a long time. BCT has enticed several countries to identify the root causes of the problems of current healthcare systems and to work on potential BC-based remedies. One of the key challenges frequently reported in the healthcare literature is data management (Ismail et al., 2019; Pandey & Litoriya, 2020), which still suffers from the loss of diagnosis data, lack of interoperability, and the inability to preserve the confidentiality and security of patient health records. To unlock the potential of BCT, a concerted effort from governments and medical institutions has been made to fund BC research projects, contributing to the national uptake of the technology to secure health and clinical records (Rathore et al., 2020). Consequently, the incorporation of BCT has profound implications and significant potential for the healthcare industry.

The high expectations raised by BCT have resulted in an increasing number of publications investigating its importance for the healthcare field. Numerous academic researchers have investigated the possibilities and challenges that BCT provides with regard to healthcare information systems (Stafford & Treiblmaier, 2020; Hasselgren et al., 2020; Tanwar et al., 2020a; Farouk et al., 2020; Khatoon, 2020; Alam Khan et al., 2020). The accumulation of research and knowledge relating to BC applications has accelerated at an unprecedented pace. However, previous investigations are mostly qualitative and conceptual in nature and thus lack a more comprehensive literature analysis (Chukwu & Garg, 2020; Houtan et al., 2020; Ben Fekih & Lahami, 2020; Sookhak et al., 2021). As such, it is still challenging for the scholarly audience to identify the core literature, publication outlets, and influential scholars; it also remains a challenge to maintain a clear picture of the extant research and discussions surrounding BC applications in the healthcare field. Although several systematic reviews exist that thoroughly investigate the opportunities and challenges of BCT in the healthcare domain (Agbo et al., 2019; Holbl et al., 2018; Hussien et al., 2019), these publications only partially reflect the overall structure, major reflections, hotspots, and prospective research trajectories pertaining to BC in healthcare.

The paucity of review articles studying developmental trends in the BC healthcare literature with bibliometric tools represents a research opportunity for the academic community. Through the power of text mining, it possible to obtain insights into the current research state of a particular subject, its development, and research patterns while one can also visualize the intellectual structure of a discipline. Unlike conventional and systematic reviews, bibliometric reviews can overcome the shortcomings associated with limited coverage of the literature. Such reviews help to uncover the intellectual structure of BC research in the healthcare field as well as the main topics that are currently under discussion.

The major focus of this paper is to understand the intellectual knowledge structure, development trends, and research foci of the BC healthcare field through a comprehensive bibliometric analysis that uses bibliometric tools and techniques. By analyzing 626 documents selected from the Web of Science (WoS) database, we conducted an analysis of published research from 2016 to 2020, identified the knowledge structure, and captured the development of BC research in the field of healthcare. Bibliometric reviews are valuable research inputs that help to ascertain the work that has been carried out in a particular discipline, discern patterns, unravel the intellectual structure of a domain of knowledge, and reach a good understanding of the existing state of the art (Portugal Ferreira, 2011). Bibliometric reviews conduct a quantitative analysis in a particular knowledge field with the goal of investigating large amounts of academic literature as units of analysis based on several criteria and metrics, such as the number of publications, citation rates of those publications, journals, and co-authors (Benson et al., 2016).

The findings of this study will be relevant and insightful for scholars and practitioners working in the field of BC and healthcare, providing the community with additional knowledge and a sharpened understanding of the current research status as well as development patterns. The undertaking of the present study can advance BC research, promote further applications, and illuminate new directions for future BC knowledge dissemination in the healthcare sector.

The remainder of this paper is organized as follows: Section 2 details the methodology used to collect the studies and the research tools used for the analysis; Section 3 presents a descriptive analysis, including the temporal and geographical distribution of BC healthcare research; Section 4 outlines the research foci identified in the BC healthcare research, including the process of keyword retrieval and frequency counting, network generation, and the interpretation of research foci; and Sections 5 and 6 conclude the paper, highlight research contributions, and suggest future research directions.

## Methodology

### Data collection

In this study, we limited our search to journal articles and conference papers indexed in the WoS. We did not fix a time interval for locating relevant BC healthcare literature. To extract articles from the selected database, we used a search strategy that retrieves papers with titles, abstracts, or keywords that contained the following terms: blockchain AND (health* OR medic* OR biomedic* OR clinic* OR doctor* OR pharmaceutical* OR illness* OR nursing OR physician* OR hospital* OR biotechnology OR diagnos* OR insurance* OR wellness OR patient* OR therapy OR disease* OR disabilit* OR treatment OR “life expectancy” OR prescription* OR surger*). To ensure the conceptual relevance of the collected publications, the authors independently screened the title, abstract, and keywords of each document. In cases where discrepancies in the assessment occurred, the respective articles were discussed until an agreement was reached. The full content of all potentially relevant studies was then assessed by at least two authors to avoid subjective bias. We obtained 626 publications for the final analysis, consisting of 619 journal articles and seven conference papers.

### Research toolkits

Knowledge mapping is an important concept in science that is commonly used to uncover and visualize groups of similar ideas and to investigate the development status of a given field, scientific collaborations, related disciplines, research hotspots, or emerging trends (Li et al., 2017). Through knowledge mapping, researchers obtain a holistic understanding of the collective knowledge domain and employ it to visualize the search and analysis outcomes. For research fields that are advancing and accelerating at a rapid pace, which is the case with BC healthcare research, knowledge mapping is a valuable tool that can be used to capture the evolution of knowledge over time and scrutinize the expansion in knowledge generation (Chen & Liu, 2005).

The analyses were done with CiteSpace, a freely available software package that analyzes and visualizes patterns and trends in academic literature (Chen, 2004). CiteSpace enables users to perform structural and temporal analyses of a variety of networks that are based on academic publications, such as co-authorship networks, author co-citation networks, and document co-citation networks. Moreover, the software supports the development of networks that are based on different node types, such as keywords, institutions, and countries, as well as different link types, such as co-citation and co-occurrence (Chen, 2006). Additionally, we used SATI3.2 to generate a keyword co-occurrence matrix and UCINET 6.0 to subsequently convert the matrix into a keyword co-occurrence network. We also used other software tools such as Excel 2016 and HistCite in the data analysis process.

## Descriptive results

### Timeline of BC healthcare research

**Fig. 1:**
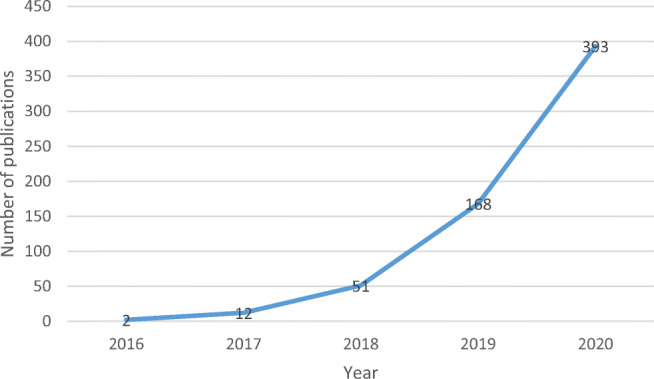
Published research on BC and healthcare

Figure 1 depicts the year-wise distribution of publications and illustrates that the initial interest in BC and healthcare emerges in the academic literature in 2016. Two articles were published in that year that launched the investigation into the potential of BCT within the field of healthcare (Yue et al., 2016; Zhang et al., 2016). Figure 1 further shows that the interest in the topic surged from 2018 onward, resulting in a total of 612 publications to date and indicating that academic scholars and practitioners view BC in healthcare as a topic worthwhile of investigation.

**Fig. 2:**
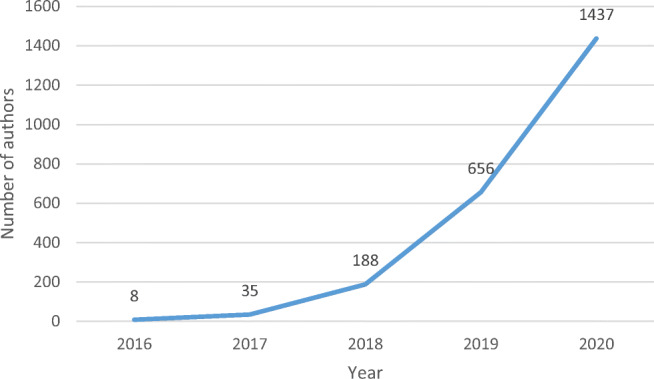
Annual distribution of the number of authors

The vigorous development of BC healthcare research has been accompanied by an increase in the annual total number of authors of publications as shown in Fig. 2. The evolution pattern of the number of authors is consistent with that of the number of publications. Only eight authors authored articles in 2016, and this number grew to 1437 in 2020. This rapid growth reflects the increased interest in BC healthcare among academic scholars.

### Journal details

Table 1 presents the top 12 journals that have published at least seven articles on BC healthcare between 2016 and 2020. These top 12 publication outlets accounted for approximately 42% of the total number of selected articles. *IEEE Access* tops the list with 100 published articles, followed by the *Journal of Medical Internet Research* and *Sensors*, which published 34 and 26 articles respectively, and *Electronics* and *Journal of Medical Systems,* which published 18 articles each. Although journals from the fields of computer science and medicine dominate the research domain, other disciplines, such as business and management, are also included in the total journal list (Abdeen et al., 2019; Hastig & Sodhi, 2020). Hence, it is recognized that BC healthcare research covers a wide knowledge base that spans academic disciplines.

**Table 1:** Journals with three or more published articles

Journal	Number of publications
IEEE Access	100
Journal of Medical Internet Research	34
Sensors	26
Electronics	18
Journal of Medical Systems	18
Sustainability	11
IEEE Internet of Things Journal	11
Applied Sciences	10
International Journal of Advanced Computer Science and Applications	10
Future Generation Computer Systems	8
Journal of Network and Computer Applications	7
IEEE Network	7

### Geographical distribution of BC healthcare research

Table 2 presents the top 12 academic institutions ranked by their number of publications on BCT and healthcare. The *University of Electronic Science and Technology of China* ranks first with 14 journal articles. 11 studies were published by *Khalifa University*, followed by 10 published studies each by *King Saud University* and *University of California San Diego*. The institutions listed in Table 2 are widely distributed geographically, which is an indicator of the global interest in BC healthcare research. The two rightmost columns in Table 2 show the citation frequency as indicated in the HistCite system. The Local Citation Score (LCS) denotes the citation frequency of a journal article, whereas the Global Citation Score (GCS) refers to the citation frequency of a specific journal article in the WOS database. Collaboration in scientific research is recognized as an effective way to build scientific capacity, advance knowledge, and share resources (Wagner et al., 2001), and the level of collaboration is considered to be one of the indexes that can be used to assess the state of research in a particular domain.

**Table 2:** Academic Institutions and the number of publications (eight or more)

Institution	Number of publications	LCS	GCS
University of Electronic Science and Technology (China)	14	146	406
Khalifa University	11	6	31
King Saud University	10	21	98
University of California San Diego	10	180	376
Chinese Academy of Sciences	9	20	112
Beijing University of Posts and Telecommunications	9	40	115
Beijing Institute of Technology	9	22	100
COMSATS University	8	1	39
Imperial College London	8	31	49
University of Texas at San Antonio	8	72	245
Deakin University	8	22	62
Qatar University	8	17	110

**Fig. 3:**
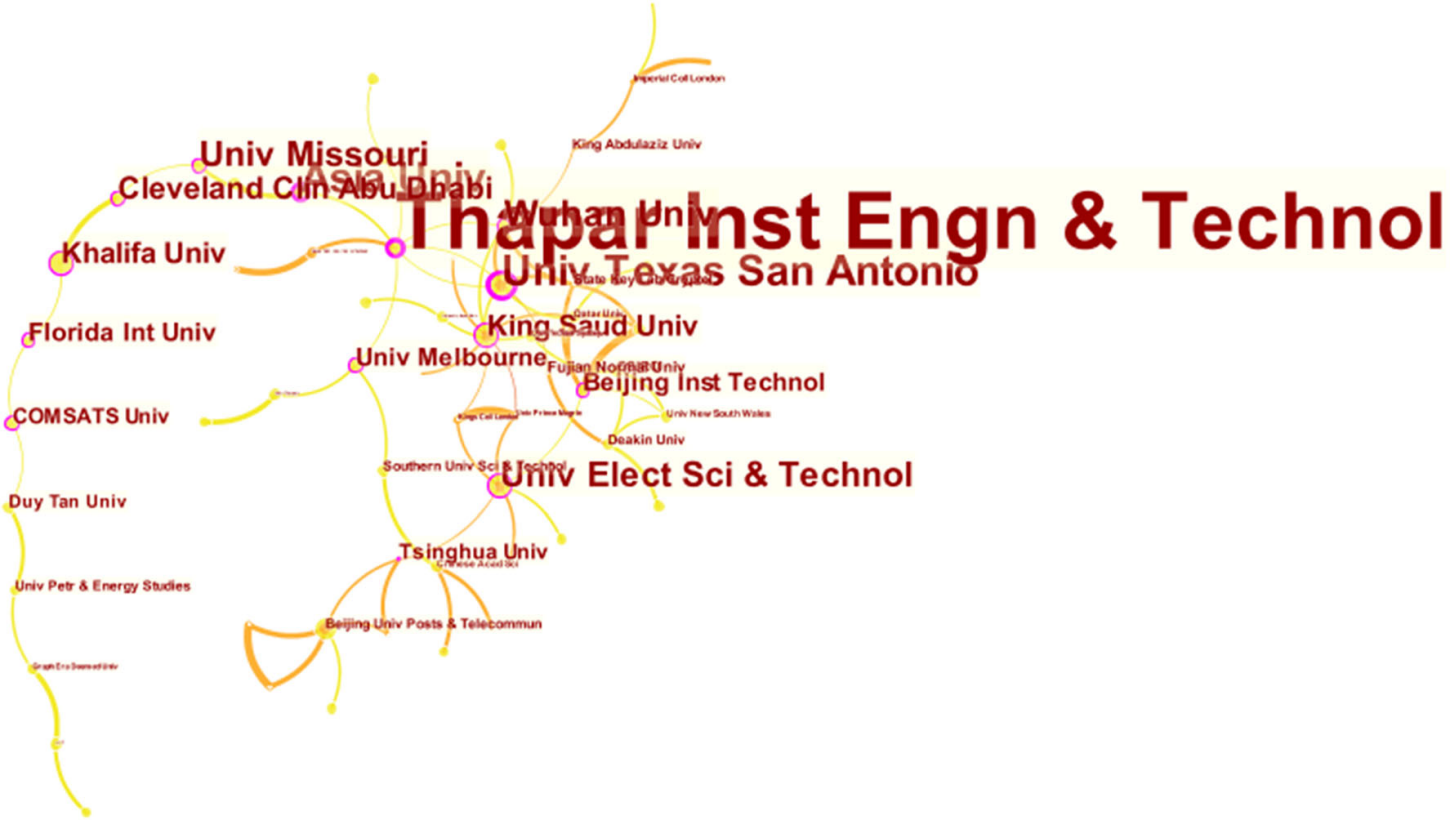
Institutional collaboration network

The institutional collaboration network, as shown in Fig. 3, illustrates the amount of collaboration in BC healthcare. Each node in the figure represents an academic institution. The size of the node is proportional to the number of journal articles published, the label font size reflects centrality (an indicator that represents the importance of a node in a network as it measures the ratio of the number of shortest paths of a given node to the total number of shortest paths between two nodes (Chen, 2005)), and the edges indicate institutional collaboration. In total, there are 100 nodes, 82 edges, and the density is 0.0166, which suggests that research collaboration across academic institutions is not prevalent.

**Fig. 4:**
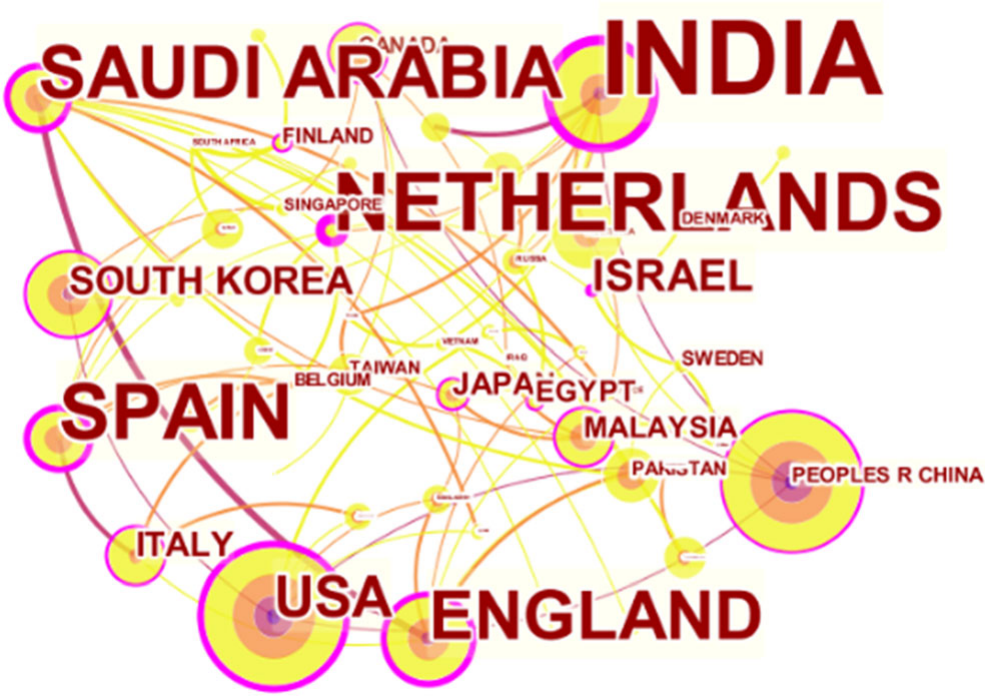
National collaboration networks

To examine research collaborations across countries, we visualized the network of national collaborations as can be seen in Fig. 4. In total, there are 49 countries with 80 national research collaboration edges.

To investigate national collaboration networks, we retrieved the necessary information related to countries with 20 publications or more as presented in Table 3. In terms of the total number of publications, China and the USA top the list with 145 articles each, followed by India and England with 74 and 68 articles, respectively. When it comes to centrality, namely, the number of times a node in a network has to be passed by, India ranks first with a ratio of 0.5, Spain is second with a ratio of 0.38, and Saudi Arabia is in the third position with a ratio of 0.36 and 33 journal articles. These countries are represented by nodes that are marked with purple rings in Fig. 4. According to the centrality index, India and England have comparative advantages in terms of publication counts and influential positions. Most of the researchers from top countries on the list started publishing in 2018 and 2019.

**Table 3:** Countries with 20 publications or more in the BC healthcare research

Country	Number of publications	Centrality	Year
People’s Republic of China	145	0.11	2016
USA	145	0.28	2017
India	74	0.5	2018
England	68	0.34	2018
South Korea	52	0.19	2018
Australia	40	0.05	2019
Canada	35	0.11	2018
Saudi Arabia	33	0.36	2018
Italy	32	0.17	2018
Pakistan	29	0.09	2019
United Arab Emirates	24	0.01	2019
Malaysia	21	0.14	2019
Spain	20	0.38	2018

### Knowledge base of BC healthcare research

As highlighted by Chen (Chen, 2006), the knowledge or intellectual base of a research front represents the citation trails of the research front in the literature. To study the knowledge base and its projected development in the BC healthcare literature, we applied a document co-citation analysis and generated a co-citation network. Co-citation networks and knowledge maps capture any two papers that are cited by a third paper or by several different papers at the same time (Small, 1973). The higher the likelihood that two documents are cited together in reference lists of other documents, the more likely it is that they have something in common. Research papers that are frequently cited together gradually garner the recognition of the scientific community and belong to a certain scientific paradigm. By analyzing a document co-citation network, it is possible to recognize and visualize the knowledge base of a field and the intellectual structure and theoretical development of existing research (Zupic & Čater, 2015).

**Fig. 5:**
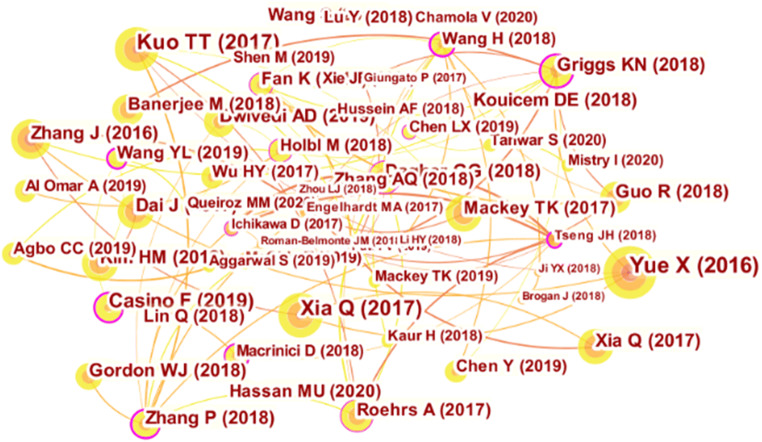
Document co-citation network

Figure 5 shows the document co-citation network for BC and healthcare. Each node in the figure reflects a cited document, and the link between two nodes represents the co-citation relationship, wherein thicker links suggest stronger relationships. The article entitled “Healthcare Data Gateways: Found Healthcare Intelligence on Blockchain with Novel Privacy Risk Control,” which was published by Yue (2016) (Yue et al., 2016) in the Journal of Medical Systems, was cited 296 times and is strongly cross-connected with Zhang et al., (2016). Figure 5 also reveals a conceptual relatedness between Yue et al., (2016) and Xia et al., (2017a). An article published in IEEE Access by Xia et al., (2017a) titled “MeDShare: trust-less medical data sharing among cloud service providers via Blockchain” was cited 205 times and is strongly connected with Xia et al., (2017b) and Yue et al., (2016). This article has a research theme that is closely related to the topics of the articles published by Xia et al., (2017b) and Yue et al., (2016). An article published by Kuo et al., (2017) entitled “Blockchain Distributed Ledger Technologies for Biomedical and Health Care Applications” in the Journal of the American Medical Informatics Association was cited 204 times. Our co-citation network of BC healthcare literature reveals that this emerging area is well formed, reflecting sufficient integration and the gradual development of the field.

**Fig. 6:**
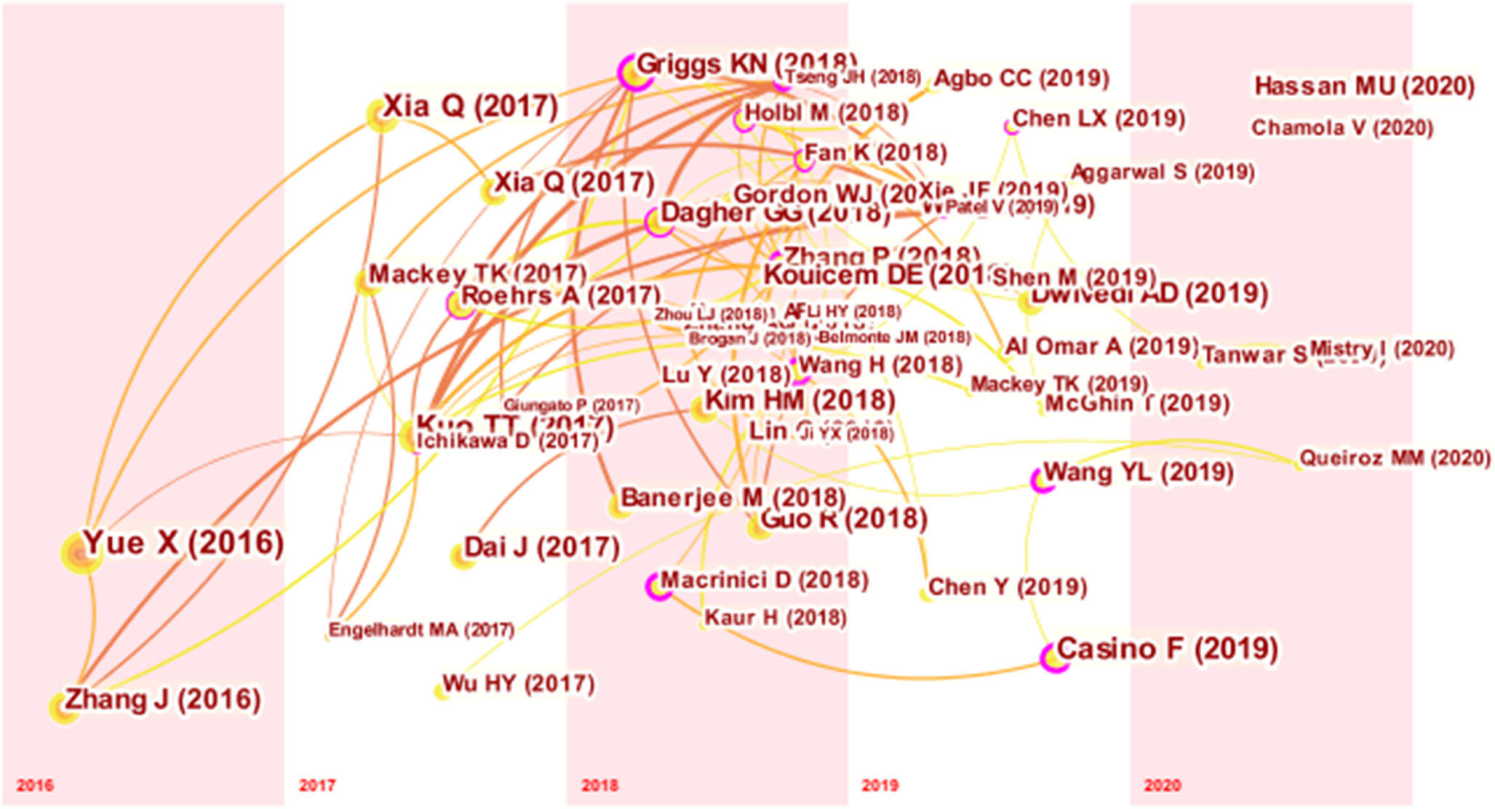
Co-citation time chart

The present study applies intermediary centrality to identify the most important studies in the field. The key scientific studies on BC healthcare research in each period are shown in Fig. 6, and they also illustrate the evolutionary research trend of the field. In addition, using CiteSpace’s citation bursts, which illustrate the most active areas of research, we found that the studies from Zhang et al., (2016) (A Secure System For Pervasive Social Network-Based Healthcare) and Yue et al., (2016) (Healthcare Data Gateways: Found Healthcare Intelligence on Blockchain with Novel Privacy Risk Control) marked the historical beginning of BCT research in the healthcare sector (see Fig. 6). Both articles were published in computer science and information systems journals with high impact factors, namely, *IEEE Access* and the *Journal of Medical Systems*. Consequently, they attracted a lot of attention from the academic community.

Table 4 presents further details regarding the nodes with a centrality greater than 0.20 in the article co-citation network. The five top articles from Wang and Song, (2018), Tseng et al., (2018), Macrinici et al., (2018), Zhang et al. (2018a), and Casino et al. (2019) have gained significant recognition in the BC healthcare literature. The lead authors of these articles have been involved in the study of BC applications from a health informatics perspective. For example, Wang was affiliated with Shandong Normal University in China and specialized in big data, artificial intelligence, the internet of things (IoT), and health informatics, whereas Tseng worked at the Taiwan Food and Drug Administration. Macrinici was affiliated with Örebro University and specialized in computer engineering, information systems, and business intelligence as is the case with Zhang who worked at Vanderbilt University in the USA and also Casino from the University of Piraeus. Several of these researchers are at a relatively early stage of their academic career, which is consistent with the research by Durach et al., (2020) who pointed out that research on the latest BC-based technologies is frequently led by younger people, including as an example Vitalik Buterin, the founder of Ethereum. This finding also illustrates that BCT strongly appeals to young scholars. Therefore, these researchers’ efforts and interest in BCT have contributed to the formation of the knowledge base of BC healthcare research, paving the way for their successors to advance the progress of BC research in the medical sector.

**Table 4:** Nodes in the article co-citation network with a centrality greater than 0.20

Author(s)	Article title	Year	Source	Citation counts	Centrality
Wang and Song, (2018)	Secure Cloud-Based EHR System Using Attribute-Based Cryptosystem and Blockchain	2018	Journal of Medical Systems	46	0.38
Tseng et al., (2018)	Governance on the Drug Supply Chain via Gcoin Blockchain	2018	International Journal of Environmental Research and Public Health	19	0.38
Macrinici et al., (2018)	Smart Contract Applications within Blockchain Technology: A Systematic Mapping Study	2018	Telematics and Informatics	41	0.29
Zhang et al., (2018a)	FHIRChain: Applying Blockchain to Securely and Scalably Share Clinical Data	2018	Computational and Structural Biotechnology Journal	81	0.28
Casino et al. (Casino et al., 2019)	A Systematic Literature Review of Blockchain-Based Applications: Current Status, Classification and Open Issues	2019	Telematics and Informatics	129	0.25
Griggs et al., (2018)	Healthcare Blockchain System Using Smart Contracts for Secure Automated Remote Patient Monitoring	2018	Journal of Medical Systems	83	0.23
Wang et al., (2019)	Making Sense of Blockchain Technology: How Will It Transform Supply Chains?	2019	International Journal of Production Economics	62	0.21

## BC healthcare research foci

Kuhn, (2012) pointed out that the development of a scientific field goes through a gradual process that gives rise to new sets of theoretical, conceptual, and methodological grounds. Along with the introduction of a new paradigm, the vocabulary with which academia interprets a new phenomenon differs. As a result, changes in vocabulary are a critical part of the scientific revolution that brings about a new phase of normal research. By measuring the frequency of keywords in the BC healthcare literature, we can therefore predict the relationship between keyword units and research foci of a specific knowledge domain during a given period. Hence, keyword co-occurrence can provide a clear idea of the research structure and focus of a specific domain. The origin of keyword co-occurrence was introduced by Callon et al., (1986) and was later widely used in the computer science field. Keyword co-occurrence is a bibliometric technique that detects the association of two terms that address a research theme in a specific knowledge domain and appear together in the same database record (i.e., the reviewed articles). The more often two keywords co-occur, the stronger the relationship between the scientific terms is. Therefore, to determine research foci in the BC healthcare literature, we adopted the keyword co-occurrence technique and followed three main steps to achieve this goal: extraction of keywords, construction of a keyword co-occurrence matrix, and analysis of data.

### Extraction of keywords and frequency counting

To reflect the emerging research hotspots, the analysis of keywords elucidates the key points discussed in the literature and acts as an important metric in quantitative studies. As a result, we used the Statistical Tool for Informatics (SATI3.2) to evaluate all selected papers, extract the top 52 keywords, and then prepare the keywords for generating a keyword co-occurrence network that serves as the foundation for further analysis. We initially pre-processed the keywords and removed inconsistencies, words compounds, and word redundancies, such as “blockchain” “blockchain technology”, or “IoT” and “Internet of Things.”

Table 5 shows the 52 extracted keywords, each with an occurrence of nine or more times, all of which represent critical topics in the BC healthcare literature. Electronic Health Records (EHRs), IoT, and smart contracts are considered to be key enablers of digital transformation in the healthcare sector (Griggs et al., 2018; Jayaraman et al., 2019). Their effective integration with BCT paves the way for a more secure platform to exchange medical information, leading to an increased sense of user data empowerment (Rahman et al., 2019; Dimitrov, 2019). Most noteworthy is the frequent mentioning of privacy, indicating that numerous scholars are actively investigating the potential of BCT to resolve challenges associated with access control and the handling of sensitive data (Nguyen et al., 2019; Thwin & Vasupongayya, 2019). The keywords in Table 5 thus illustrate that it is not only the potential of BCT that attracts researchers, but also the various obstacles that need to be overcome prior to its introduction. Apart from privacy issues, this also includes the interoperability of legacy systems with BCT (i.e., the ability to BC-based systems to communicate with each other) and the manifold problems related to data security. In this regard, BC can alleviate several problems, but might also open up new attack vectors (Farouk et al., 2020).

**Table 5:** Keyword frequency (>8)

No.	Keyword	Freq.
1	Blockchain	516
2	IoT*	115
3	Smart Contract	101
4	Security	74
5	Healthcare	68
6	EHR	66
7	Privacy	59
8	DLT	57
9	Ethereum	30
10	Cloud Computing	29
11	Bitcoin	28
12	eHealth	24
13	ML	24
14	Data Sharing	23
15	Interoperability	22
16	Access Control	21
17	AI	21
18	Medical Services	19
19	Authentication	18
20	Cryptocurrency	18
21	Privacy Preserving	18
22	SC	17
23	Smart Cities	17
24	Big Data	15
25	COVID-19	15
26	Cryptography	15
27	Data Privacy	15
28	Industry 4.0	15
29	Consensus	14
30	SCM	14
31	Edge Computing	13
32	Insurance	13
33	Medical Diagnostic Imaging	13
34	PHR	13
35	Industries	12
36	CPSs	11
37	Data Security	11
38	Decentralization	11
39	HIE	11
40	IIoT	11
41	Medical Data	11
42	mHealth	11
43	Traceability	11
44	Fog Computing	10
45	IPFS	10
46	ABE	9
47	Contracts	9
48	Encryption	9
49	Hyperledger Fabric	9
50	Sensors	9
51	Sustainability	9
52	Trust	9

* IoT: Internet of Things/ EHR: Electronic Health Records/ DLT: Distributed Ledger Technology/ ML: Machine Learning/ AI: Artificial Intelligence/ SC: Supply Chain/ SCM: Supply Chain Management/ PHR: Personal Health Record/ CPSs: Cyber Physical Systems/ HIE: Health Information Exchange/ IIoT: Industrial Internet of Things/ IPFS: Inter-Planetary File System / ABE: Attribute-Based Encryption

### Keyword co-occurrence and analysis of research foci

**Fig. 7:**
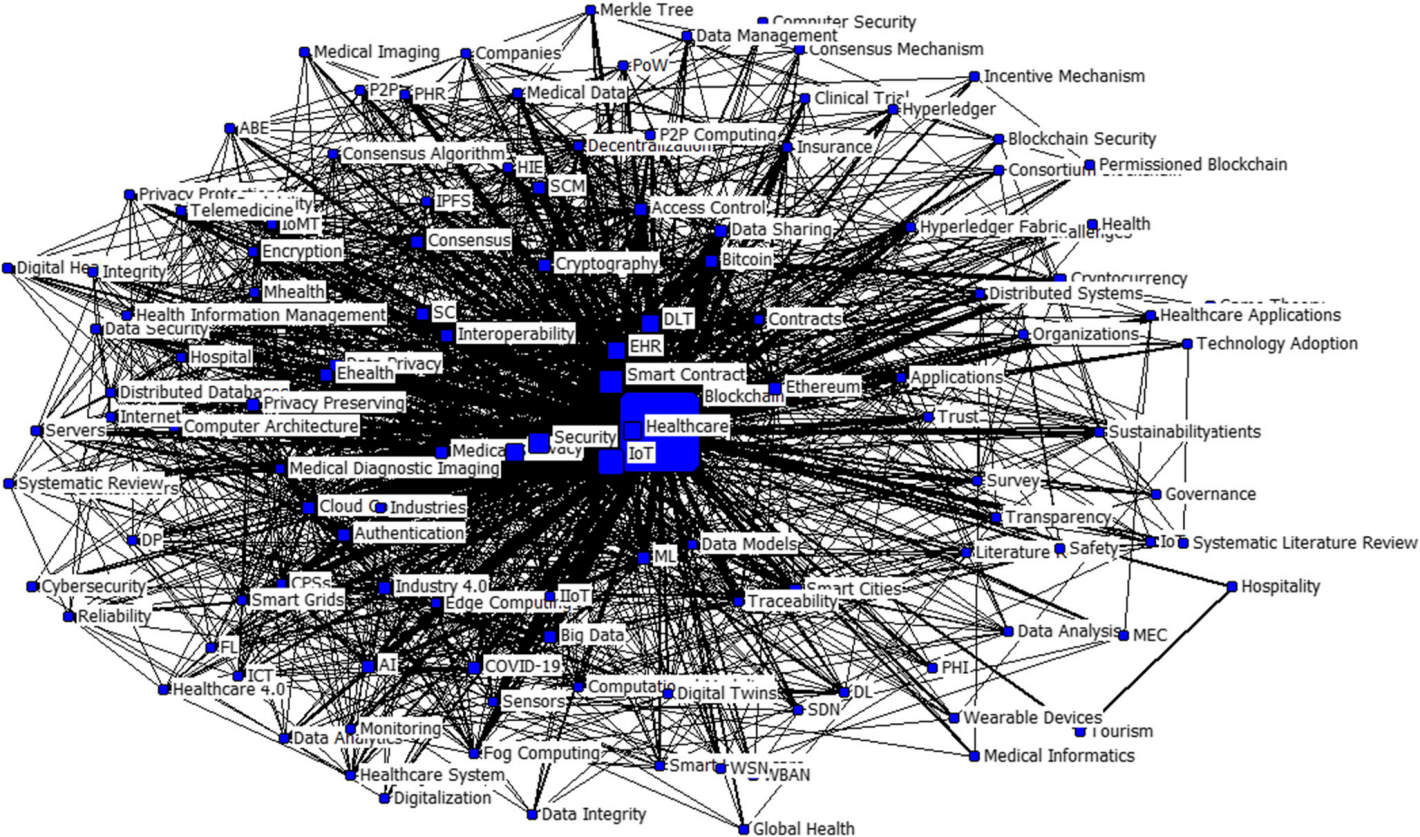
Keyword co-occurrence network

We used SATI3.2 to build a 127*127 co-keyword matrix as is shown in Table 6. We employed UCINET 6.698 to convert the format of the co-keyword matrix and then generate the visualization of the keyword co-occurrence network as depicted in Fig. 7 below. As can be seen from the map, the nodes are the keywords, and their size reflects the different levels of betweenness. The bigger the nodes are, the greater the betweenness of the keywords is. Similarly, the greater the central position of the nodes, the more probable it is that the keywords are the main focus in BC healthcare research. The edges linking the nodes represent the co-occurrence frequency of two different keywords. The thicker the edges are, the greater the co-occurrence of the keywords and the closer the relationship between them is.

**Table 6:** Co-keyword matrix (excerpt)

	Blockchain	IoT	Smart Contract	Security	Healthcare	EHR	Privacy	DLT	Ethereum	Cloud Computing	Bitcoin	eHealth	ML	…
Blockchain	516	97	95	66	66	58	52	54	30	22	25	21	18	…
IoT	97	115	16	23	23	4	16	5	4	9	5	4	5	…
Smart Contract	95	16	101	11	14	6	11	11	19	1	8	2	4	…
Security	66	23	11	74	14	10	32	5	2	6	1	4	3	…
Healthcare	66	23	14	14	68	8	8	11	8	4	2	2	3	…
EHR	58	4	6	10	8	66	8	3	1	6	0	5	1	…
Privacy	52	16	11	32	8	8	59	2	1	5	2	2	5	…
DLT	54	5	11	5	11	3	2	57	2	0	5	4	0	…
Ethereum	30	4	19	2	8	1	1	2	30	0	7	0	2	…
Cloud Computing	22	9	1	6	4	6	5	0	0	29	0	1	3	…
Bitcoin	25	5	8	1	2	0	2	5	7	0	28	0	1	…
eHealth	21	4	2	4	2	5	2	4	0	1	0	24	0	…
ML	18	5	4	3	3	1	5	0	2	3	1	0	24	…
Data Sharing	18	2	2	3	3	8	6	3	0	3	0	1	0	…
Interoperability	21	4	3	5	4	4	2	6	1	2	0	1	0	…
Access Control	20	5	5	3	3	4	5	2	3	3	0	1	0	…
AI	15	6	1	3	1	2	3	0	0	4	0	0	6	…
Medical Services	17	7	4	5	3	4	4	2	1	6	0	1	1	…
…	…	…	…	…	…	…	…	….	…	…	…	…	…	…

Figure 7 illustrates that blockchain, EHRs, smart contracts, healthcare, IoT, privacy, and security are the focal points in current BC healthcare research. In recent years, the healthcare sector has witnessed the proliferation of EHRs and a paradigm shift in the ways that medical data is stored and exchanged among patients and healthcare providers (Nguyen et al., 2019). Patient-centric care can be improved and supported by facilitated access to real-time information through EHR. However, the benefits of EHR might be nullified by increasing concerns about medical data privacy and security issues of e-health systems. To overcome these challenges, BCT makes EHR data more easily manageable and transferable among patients and healthcare entities (Griggs et al., 2018). BCT enables patients to have comprehensive, immutable records and to access EHRs independent of healthcare providers and treatment websites (Guo et al., 2018). Instead of being scattered across different healthcare organizations, the transition of EHR on BCs enables users to achieve higher operational efficiencies (Zhou et al., 2019; Daraghmi et al., 2019; Pirtle & Ehrenfeld, 2018), ensures more accountability for data management between the users, creates opportunities for secure healthcare data trading (Hasan et al., 2019; Li et al., 2019), and increases the interoperability of data between different healthcare providers (Hussien et al., 2019; Mayer et al., 2019).

Another capability of the BC ecosystem lies in smart contracts. The concept of a smart contract was popularized by Nick Szabo (Szabo, 1996) to assist users in digitally formalizing and securing relationships over a network (Szabo, 1997). Smart contracts are developed to follow rules that are programmatically encoded on the BC (Randall et al., 2017). With the help of these tools, BCT can interact with the various demands of the healthcare system (Daraghmi et al., 2019), eliminate the reliance on central servers, reduce transaction costs (Hussien et al., 2019; Casino et al., 2019), enforce access control and auditing on EHRs (Yang et al., 2019), and integrate new medical technologies (Pandey & Litoriya, 2020; Roehrs et al., 2017).

Smart contracts have been heralded as so-called killer applications in IoT, facilitating tasks such as the execution of automated reliable transactions, payment, and fee collection (Syed et al., 2019). The combination of BC-connected medical wearables and IoT is expected to heighten the level of engagement experienced by patients, providing them with an increased sense of data control and security (Silva et al., 2019). Using BCT and smart contracts, IoT can support real-time communication, instant patient monitoring and medical interventions, and preventive medical services (Griggs et al., 2018). By leveraging a healthcare-focused version of the IoT, which Ahmed, (2019) calls the Internet of Medical Things (IoMT), it is possible for caretakers to assess the health situation of patients without the need for frequent hospital consultations. Importantly, data gathered by body sensors worn by patients, such as heart rate or blood content monitors, can be secured in the BC network (Shuaib et al., 2019). The rise of IoMT brings about new types of medical practices that can be greatly supported by BCT, resulting in more patient-driven processes and practical insights for healthcare personnel.

The “blockchain” node in the center of Fig. 7 has a close connection with IoT, smart contracts, and security. The tight relationship between BC and these technologies indicates promising technological combinations for healthcare. More specifically, BCT ensures a high level of security and privacy while operating with EHR, smart contracts, and the IoT. With the increasing requirement for more secure healthcare data management systems, interoperability, and patient-centered care, BC is becoming a powerful foundational technology.

While current BC use cases hint at innovative solutions for decentralized healthcare data storage (Gürsoy et al., 2020), several challenges and obstacles remain unresolved, thereby hampering its effective integration. For instance, Gordon and Catalini, (2018) point out that BC adoption in healthcare is heavily dependent on the openness, interest, and willingness of all stakeholders to engage and trust the technology and its value. Similarly, Hoy, (2017) argues that it is imperative to raise awareness for BC and educate users on the benefits and usage of the technology to drive adoption and expand its applicability in healthcare. Chavali et al., (2018) point out that healthcare providers need to develop a shared approach for the storage of medical data to ensure data integration and interoperability with existing systems. Accordingly, the high complexity as well as a lack of education and understanding of how BC works may pose a barrier for further adoption and usage (Boulos et al., 2018). Resistance to innovation and a viable interest in keeping the status quo may hinder BC’s progress in healthcare. Furthermore, some individuals distrust the technology, while others overuse the term without delivering value, creating a false impression of how BC could benefit healthcare (Randall et al., 2017).

From a technical perspective, multiple parameters play a key role in determining the performance of BC-based healthcare systems. For example, the use of the Bitcoin BC in healthcare is not practical due to its high energy consumption, limited scalability, and low transaction throughput (Ismail et al., 2019). As medical data and the number of network participants (e.g., patients, healthcare institutions) increase, applications enabled by BC will become significantly more challenging to run (Hussien et al., 2019). In this regard, Shuaib et al., (2019) state that, at some point, computational resources, such as processing power and storage mediums, will be incapable of satisfying the needs of network users, thereby causing a latency of data transmission. Moreover, despite the importance of decentralization in ensuring data security and privacy, BC is still subject to vulnerabilities and cyberattacks, such as leakage of sensitive data, theft of private keys, and majority attacks (Tanwar et al., 2020b). Such attacks could compromise the consensus protocol and lead to the unintended access and manipulation of healthcare data.

From a regulatory perspective, Engelhardt, (2017) adds that the unclear legal and regulatory landscape of BC in healthcare creates insecurity regarding the future standing of any applications. Likewise, Boulos et al., (2018) point out that certain business models in the healthcare market do not reflect the real needs of the stakeholders; therefore, it is essential to design viable and sustainable business models that build on the technology’s value. Additionally, low academic interest and a lack of research hinders the critical evaluation of the fundamental problems involved with BC that may solve and stall the technology’s development and adoption (Till et al., 2017). Without a supportive regulatory environment and concerted research efforts, it remains unclear how BC will evolve in the future and how healthcare data will be protected against misuse and privacy violations. As BC has not reached a state of maturity and many aspects of the technology are yet to be improved, it is argued that security, privacy, usability, and architecture design are crucial prerequisites for broader technology acceptance and adoption in healthcare in the future (Gordon & Catalini, 2018). Overall, the challenges of BC in the healthcare sector can be summarized into scalability issues, privacy and security concerns, system complexity, technological immaturity, resistance to change, and lack of supportive regulations.

## Conclusions and limitations

The introduction of BCT constitutes a major shift in the healthcare sector, offering a wide range of innovative applications and use cases that also lead to new challenges. The emergence of BC provides unprecedented opportunities and capabilities that can be leveraged to overcome numerous challenges that the healthcare sector currently faces. BCT can help to achieve a sustainable healthcare ecosystem through improved security, confidentiality, data integrity, and also disintermediation. The tamper-proof recording mechanism of BC makes it the first choice to build highly resilient healthcare data systems that can simultaneously secure patient data and preserve privacy. Operating a BC platform can help to mitigate inefficient practices and recurrent data breaches that often plague the healthcare sector (Farouk et al., 2020). Moreover, BC secures and optimizes the process of sharing EHRs, facilitates drug traceability, and closely integrates IoT devices used in the healthcare network. In this regard, the MedRec protocol from MIT Examples illustrates the importance of BC for ensuring a high level of data exchange and interoperability (Ekblaw, 2016), IBM initiates Hyperledger project to promote the application of BC to healthcare and IoT (Griggs et al., 2018), and Deloitte and Accenture develop BC prototypes to store healthcare data and manage EHRs (Kuo et al., 2017).

In the present study, we conducted a bibliometric analysis of current BC healthcare research. The main goal of this study was to review BC research in the healthcare field through the bibliometric analysis of 626 studies. Initially, we carried out an advanced search of the WOS database and manually screened all of the articles for relevance. Then, the time, journal, and space distribution of publications were analyzed to reveal the evolutionary pattern of BC healthcare research and identify the main journals in the field as well as the geographical locations of the respective research hotspots. Additionally, the networks of institutional collaboration, national collaboration, co-cited journal articles, and keyword co-occurrence were analyzed to better understand the overall situation of BC healthcare research.

In summary, the results from our study clearly indicate that research in BC healthcare has considerably increased in recent years. The number of scholars working in the BC healthcare research field is rapidly increasing. Furthermore, we identified the dominant journals that published BC healthcare research between 2016 and 2020, including *IEEE Access, Sensors, Electronics,* and numerous medical journals, such as the *Journal of Medical Internet Research* and *Journal of Medical Systems*. When it comes to leading institutions, the *University of Electronic Science and Technology of China* was the most productive institution in this area, and strong research collaboration networks exist between numerous institutions. In order to further develop this highly relevant field, we recommend that collaboration among authors from different academic institutions be fostered by launching joint research projects and integrating researchers from developing countries. As stated by Leino-Kilpi et al., (2003), international research collaboration is valuable for producing fresh ideas for new projects. Collaboration helps to bring together diverse skills and scientific thoughts and serves as an effective channel for providing access to scientific knowledge and technologies for developing and newly-developed countries (Kim, 2006). As a result, we also recommend institutional collaboration to substantiate the overall research strength and use resources more efficiently.

From the perspective of national contributions, China and the USA made the most contributions in the academic BC healthcare field. Of course, this only pertains to publications in the English language that provided the foundation of this literature review. Additionally, we found a considerable number of publications from India and England. The leading publications in this field garner a lot of attention from the scientific community and contribute to the development of the BC paradigm in the healthcare sector. Generally, collaborative research arrangements and ties have been developed over time among nations, illustrating that BC healthcare research has spread rapidly and covers a lot of related topics.

Concerning the knowledge base, the present study identified the outstanding scholars and core content of the BC healthcare research in recent years. These researchers have significantly contributed to the conceptual development of BC healthcare, touched on several issues plaguing the medical sector, and demonstrated the effectiveness and efficiency of operating within a BC environment. Accordingly, we managed to identify several core themes based on the used keywords, ranging from the role of BCT to support the security and privacy of EHRs, smart contacts, and data generated from medical IoT devices to the facilitation of data exchange, interoperability, financial transactions, and traceability of medicines and patient-sensitive data. The set of generated keywords helps to capture the core content of the BC healthcare research and highlights the focal topics that represent this area of scientific knowledge.

Although the analysis of keywords provides valuable information for scholars and practitioners working in the healthcare community, the use of the WOS database does not guarantee a comprehensive coverage of BC healthcare research, which also includes publications that did not fulfill our selection criteria, most notably the use of the English language. However, since WOS is considered to be the most authoritative source of data for many scientific studies, the analysis of BC healthcare literature gives us sufficient confidence in the quality and reliability of our findings. Similarly, we encourage future research in this direction to use comprehensive samples of data collected from various and comprehensive academic databases, such as Scopus and Google Scholar. The incorporation of other types of publications, such as books, chapters, reports, and practitioner magazines, may also provide a more comprehensive and timely analysis of BC healthcare research.

## Future research directions

BCT has emerged as a foundational innovation with strong implications for the economy and society, including the healthcare sector. It represents an entirely new approach to manage healthcare-related information in a secure, efficient, and scalable manner. The capabilities of BC are expected to optimize healthcare processes, increase patients’ control over medical data, and ultimately improve the overall outcome of healthcare services. The promising opportunities of the technology are numerous, ranging from the efficient handling of healthcare data, the prevention of privacy breaches, the enhancement of interoperability, better delivery of medical treatments, and the traceability of medicines and prescriptions to the increased control of the pharmaceutical supply chain and all IoT devices used. However, the benefits of BCT also yield various challenges and obstacles that need to be addressed prior to a wide-scale implementation of the technology. For example, the technical issues related to BC include limited scalability (Ismail et al., 2019; Dwivedi et al., 2019; Lee & Yang, 2018), security threats (Rahmadika & Rhee, 2019), high energy consumption in public and permissionless networks (Ismail et al., 2019; Monrat et al., 2019), increased complexity (Kumar et al., 2019; Li et al., 2018), and data redundancy. With an increasing number of nodes constituting the network and participating in the mining processes, the number of transactions and message transfers increases, which results in limited scalability (Ismail et al., 2019). Therefore, the introduction of more scalable BCs is imperative, and research addressing these issues is needed. Future studies may examine the parameters determining the scalability of BC healthcare systems, such as block propagation rate, consensus mechanism, transaction throughput, and the size of blocks used.

Although BCT helps to ensure the security of the patient’s medical data, BC systems are not immune against cyberattacks. For example, the use of public BC networks makes patient data susceptible to privacy intrusions as linking data could reveal the owners and their personal information (Radanovic & Likic, 2018). The loss of a private key could cause a total loss of control over the stored information. Therefore, research on improving the security and usability of BC-based health systems is needed to secure data storage, reduce privacy violations, and prevent malicious attacks. Similarly, the use of more energy-efficient and scalable BC systems should not be neglected. So far, a major criticism of public and permissionless BC networks pertains to their energy-demanding nature, high electricity consumption, and computational resources (Hasselgren et al., 2020). Research should be devoted to increasing the efficiency of BC in handling large healthcare datasets at a low level of latency. BC-based healthcare systems should be designed in a way that simplifies the retrieval of medical information and the management of records.

Several stakeholders are reluctant to host BC in the healthcare sector (Stafford & Treiblmaier, 2020). Physicians are hesitant to embrace the technology and healthcare service providers are disinclined toward the exchange of data based on the perception that health regulations prevent such sharing even in an anonymized form (Zhang et al., 2018b). Furthermore, the adoption of BC necessitates a substantial investment in additional capabilities and resources, such as training, human resource development, and IT infrastructure as well as the ability to adapt to a new modus operandi. The lack of a legal framework for the interoperability of electronic healthcare data and the unclear legal liability in the case of medical errors, privacy violations, and security issues also constitute major barriers to BC adoption. Therefore, regulators need to closely investigate this issue to leverage BC implementations in order to improve patient services and promote sustainable health systems. Research on how regulators and lawmakers could transform the existing legal obligations into the BC system is imperative to ensure the consistency of legal language and to strive to eliminate ambiguity when rewriting legal rules as codes in the BC. Furthermore, patients might be discouraged and avail themselves of reaping the benefits associated with their participation in a BC-healthcare system. In this regard, future research should focus on studying patient acceptance of BC, the expectations of users, and the determinants of their satisfaction. Addressing these points will also benefit practitioners who design patient-centric BC healthcare systems.

## References

[CR1] Abdeen MAR, Ali T, Khan Y, Yagoub MCE (2019). Fusing identity management, HL7 and Blockchain into a global healthcare record sharing architecture. Int J Adv Comput Sci Appl.

[CR2] Agbo CC, Mahmoud QH, Eklund JM (2019) Blockchain Technology in Healthcare: A Systematic Review. Healthcare 7. 10.3390/healthcare7020056 10.3390/healthcare7020056 PMC6627742 30987333

[CR3] Ahmed M (2019). False image injection prevention using iChain. Appl Sci.

[CR4] Alam Khan F, Asif M, Ahmad A, Alharbi M, Aljuaid H (2020). Blockchain technology, improvement suggestions, security challenges on smart grid and its application in healthcare for sustainable development. Sustain Cities Soc.

[CR5] Alkhaldi N. (2020) Blockchain in healthcare: use cases for your practice. https://www.itransition.com/blog/blockchain-in-healthcare (accessed May 14, 2020)

[CR6] Ben Fekih R, Lahami M, Jmaiel M, Mokhtari M, Abdulrazak B, Aloulou H, Kallel S (2020). Application of Blockchain Technology in Healthcare: a comprehensive study. Impact digit. Technol. Public health Dev. Dev. Ctries.

[CR7] Benson MH, Lippitt CD, Morrison R, Cosens B, Boll J, Chaffin BC, Fremier AK, Heinse R, Kauneckis D, Link TE, Scruggs CE, Stone M, Valentin V (2016). Five ways to support interdisciplinary work before tenure. J Environ Stud Sci.

[CR8] Boulos MNK, Wilson JT, Clauson KA (2018) Geospatial blockchain: promises, challenges, and scenarios in health and healthcare. Int. J. Health Geogr. 17. 10.1186/s12942-018-0144-x 10.1186/s12942-018-0144-x PMC6033217 29973196

[CR9] Callon M, Law J, Rip A, Callon M, Law J, Rip A (1986). How to study the force of science. Mapp.

[CR10] Carpio, Blockchain in Healthcare: Complex Challenges, Overshadowed by the Hype, Need to be Overcome, DataArt Website. (2018). https://www.dataart.com.ar/news/blockchain-in-healthcare-complex-challenges-overshadowed-by-the-hype-need-to-be-overcome/ (accessed May 14, 2020)

[CR11] Casino F, Dasaklis TK, Patsakis C (2019). A systematic literature review of blockchain-based applications: current status, classification and open issues. Telemat. Inform..

[CR12] Chavali LN, Prashanti NL, Sujatha K, Rajasheker G, Kavi Kishor PB (2018). The emergence of blockchain technology and its impact in biotechnology, pharmacy and life sciences. Curr Trends Biotechnol Pharm.

[CR13] Chen C (2004). Searching for intellectual turning points: progressive knowledge domain visualization. Proc Natl Acad Sci.

[CR14] Chen C (2005) The centrality of pivotal points in the evolution of scientific networks, in: Proc. 10th Int. Conf. Intell. User interfaces, Association for Computing Machinery. pp. 98–105. 10.1145/1040830.1040859

[CR15] Chen C (2006). CiteSpace II: detecting and visualizing emerging trends and transient patterns in scientific literature. J Am Soc Inf Sci Technol.

[CR16] Chen Y, Liu Z (2005) The rise of mapping knowledge domain. Stud. Sci. Sci. 2

[CR17] Chukwu E, Garg L (2020). A systematic review of Blockchain in healthcare: frameworks, prototypes, and implementations. IEEE Access..

[CR18] Daraghmi E-Y, Daraghmi Y-A, Yuan S-M (2019). MedChain: a Design of Blockchain-Based System for medical records access and permissions management. IEEE Access.

[CR19] Dimitrov DV (2019). Blockchain Applications for Healthcare Data Management, Healthc. Inform. Res..

[CR20] Durach CF, Blesik T, von Düring M, Bick M (2020) Blockchain applications in supply chain transactions. J. Bus. Logist. 10.1111/jbl.12238

[CR21] Dwivedi AD, Srivastava G, Dhar S, Singh R (2019) A Decentralized Privacy-Preserving Healthcare Blockchain for IoT. Sensors 19. 10.3390/s19020326 10.3390/s19020326 PMC6359727 30650612

[CR22] Ekblaw A (2016) A. Azaria, MedRec: Medical Data Management on the Blockchain, Viral Commun. . https://viral.media.mit.edu/pub/medrec (accessed April 2, 2020)

[CR23] Engelhardt MA (2017). Hitching Healthcare to the Chain: An Introduction to Blockchain Technology in the Healthcare Sector. Technol. Innov. Manag. Rev..

[CR24] Farouk A, Alahmadi A, Ghose S, Mashatan A (2020). Blockchain platform for industrial healthcare: vision and future opportunities. Comput Commun.

[CR25] Gordon WJ, Catalini C (2018). Blockchain Technology for Healthcare: facilitating the transition to patient-driven interoperability. Comput. Struct. Biotechnol. J..

[CR26] Griggs KN, Ossipova O, Kohlios CP, Baccarini AN, Howson EA, Hayajneh T (2018). Healthcare Blockchain system using smart contracts for secure automated remote patient monitoring. J Med Syst.

[CR27] Guo R, Shi H, Zhao Q, Zheng D (2018). Secure attribute-based signature scheme with multiple authorities for Blockchain in electronic health records systems. IEEE Access..

[CR28] Gürsoy G, Bjornson R, Green ME, Gerstein M (2020). Using blockchain to log genome dataset access: efficient storage and query. BMC Med Genet.

[CR29] Hanley BP (2018) The false premises and promises of Bitcoin, ArXiv13122048 Cs Q-fin. http://arxiv.org/abs/1312.2048 (accessed March 28, 2020)

[CR30] Hasan K, Biswas K, Ahmed K, Nafi NS, Islam MS (2019). A comprehensive review of wireless body area network. J Netw Comput Appl.

[CR31] Hasselgren A, Kralevska K, Gligoroski D, Pedersen SA, Faxvaag A (2020). Blockchain in healthcare and health sciences-a scoping review. Int J Med Inf.

[CR32] Hastig GM, Sodhi MS (2020). Blockchain for supply chain traceability: business requirements and critical success factors. Prod Oper Manag.

[CR33] Holbl M, Kompara M, Kamisalic A, Zlatolas LN (2018) A Systematic Review of the Use of Blockchain in Healthcare. Symmetry 10. 10.3390/sym10100470

[CR34] Houtan B, Hafid AS, Makrakis D (2020). A survey on Blockchain-based self-sovereign patient identity in healthcare. IEEE Access..

[CR35] Hoy MB (2017). An introduction to the Blockchain and its implications for libraries and medicine. Med Ref Serv Q.

[CR36] Hussien HM, Yasin SM, Udzir SNI, Zaidan AA, Zaidan BB (2019) A Systematic Review for Enabling of Develop a Blockchain Technology in Healthcare Application: Taxonomy, Substantially Analysis, Motivations, Challenges, Recommendations and Future Direction. J. Med. Syst. 43. 10.1007/s10916-019-1445-8 10.1007/s10916-019-1445-8 31522262

[CR37] Ismail L, Materwala H, Zeadally S (2019). Lightweight Blockchain for healthcare. IEEE ACCESS.

[CR38] Jayaraman R, Salah K, King N (2019). Improving opportunities in healthcare supply chain processes via the internet of things and Blockchain technology. Int J Healthc Inf Syst Inform.

[CR39] Khatoon A (2020). A Blockchain-based smart contract system for healthcare management. Electronics..

[CR40] Kim K-W (2006). Measuring international research collaboration of peripheral countries: taking the context into consideration. Scientometrics..

[CR41] Kuhn TS (2012) The structure of scientific revolutions: 50th anniversary edition. University of Chicago Press

[CR42] Kumar A, Liu R, Shan Z (2019). Is Blockchain a silver bullet for supply chain management? Technical challenges and research opportunities. Decis Sci.

[CR43] Kuo T-T, Kim H-E, Ohno-Machado L (2017). Blockchain distributed ledger technologies for biomedical and health care applications. J Am Med Inform Assoc.

[CR44] Lee SH, Yang CS (2018) Fingernail analysis management system using microscopy sensor and blockchain technology. Int. J. Distrib. Sens. Netw. 14. 10.1177/1550147718767044

[CR45] Leino-Kilpi H, Välimäki M, Dassen T, Gasull M, Lemonidou C, Scott PA, Schopp A, Arndt M, Kaljonen A (2003). Perceptions of autonomy, privacy and informed consent in the Care of Elderly People in five European countries: comparison and implications for the future. Nurs Ethics.

[CR46] Li X, Ma E, Qu H (2017). Knowledge mapping of hospitality research − a visual analysis using CiteSpace. Int J Hosp Manag.

[CR47] Li X, Huang X, Li C, Yu R, Shu L (2019). EdgeCare: leveraging edge computing for collaborative data Management in Mobile Healthcare Systems. IEEE Access..

[CR48] Li H, Zhu L, Shen M, Gao F, Tao X, Liu S (2018) Blockchain-Based Data Preservation System for Medical Data. J. Med. Syst. 42. 10.1007/s10916-018-0997-3 10.1007/s10916-018-0997-3 29956058

[CR49] Macrinici D, Cartofeanu C, Gao S (2018). Smart contract applications within blockchain technology: a systematic mapping study. Telemat Inform.

[CR50] Mayer AH, da Costa CA, da Righi RR (2019) Electronic health records in a Blockchain: A systematic review. Health Informatics J. 10.1177/1460458219866350 10.1177/1460458219866350 31566472

[CR51] Monrat AA, Schelen O, Andersson K (2019). A survey of Blockchain from the perspectives of applications, challenges, and opportunities. IEEE Access..

[CR52] Nakamoto S (2008) Bitcoin: a peer-to-peer electronic cash system, Www.Bitcoin.Org.9. 10.1007/s10838-008-9062-0, 53, 67

[CR53] Nguyen DC, Pathirana PN, Ding M, Seneviratne A (2019). Blockchain for secure EHRs sharing of Mobile cloud based E-health systems. IEEE Access..

[CR54] Pandey P, Litoriya R (2020) Securing and authenticating healthcare records through blockchain technology. Cryptologia:1–16. 10.1080/01611194.2019.1706060

[CR55] Pirtle C, Ehrenfeld J (2018) Blockchain for Healthcare: The Next Generation of Medical Records? J. Med. Syst. 42. 10.1007/s10916-018-1025-3 10.1007/s10916-018-1025-3 30097733

[CR56] Portugal Ferreira M (2011). A bibliometric study on Ghoshal’s managing across Borders. Multinatl Bus Rev.

[CR57] Radanovic I, Likic R (2018). Opportunities for use of Blockchain Technology in Medicine. Appl Health Econ Health Policy.

[CR58] Rahmadika S, Rhee K-H (2019) Toward privacy-preserving shared storage in untrusted Blockchain P2P networks. Wirel. Commun. Mob. Comput. 10.1155/2019/6219868

[CR59] Rahman MA, Rashid MM, Le Kernec J, Philippe B, Barnes SJ, Fioranelli F, Yang S, Romain O, Abbasi QH, Loukas G, Imran M (2019) A Secure Occupational Therapy Framework for Monitoring Cancer Patients’ Quality of Life. Sensors 19. 10.3390/s19235258 10.3390/s19235258 PMC6928807 31795384

[CR60] Randall D, Goel P, Abujamra R (2017). Blockchain applications and use cases in health information technology. J Health Med Inform.

[CR61] Rathore H, Mohamed A, Guizani M (2020) A Survey of Blockchain Enabled Cyber-Physical Systems. Sensors 20. 10.3390/s20010282 10.3390/s20010282 PMC6983181 31947860

[CR62] Rejeb A, Sűle E, Keogh JG (2018). Exploring new technologies in procurement. Transp Logist Int J.

[CR63] Rejeb A, Keogh JG, Zailani S, Treiblmaier H, Rejeb K (2020). Blockchain Technology in the Food Industry: a review of potentials, challenges and future research directions. Logistics..

[CR64] Roehrs A, da Costa CA, da Righi RR (2017). OmniPHR: A distributed architecture model to integrate personal health records. J. Biomed. Inform.

[CR65] Shuaib K, Saleous H, Shuaib K, Zaki N (2019). Blockchains for secure digitized medicine. J Pers Med.

[CR66] Silva CA, Aquino GS, Melo SRM, Egídio DJB (2019). A fog computing-based architecture for medical records management. Wirel Commun Mob Comput.

[CR67] Small H (1973). Co-citation in the scientific literature: a new measure of the relationship between two documents. J Am Soc Inf Sci.

[CR68] Sookhak M, Jabbarpour MR, Safa NS, Yu FR (2021). Blockchain and smart contract for access control in healthcare: a survey, issues and challenges, and open issues. J Netw Comput Appl.

[CR69] Stafford TF, Treiblmaier H (2020). Characteristics of a Blockchain ecosystem for secure and sharable electronic medical records. IEEE Trans Eng Manag.

[CR70] Syed TA, Alzahrani A, Jan S, Siddiqui MS, Nadeem A, Alghamdi T (2019). A comparative analysis of Blockchain architecture and its applications: problems and recommendations. IEEE ACCESS..

[CR71] Szabo N (1996) Smart contracts: building blocks for digital free markets. Extropy J Transhuman Thought:1–10

[CR72] Szabo N (1997) Formalizing and securing relationships on public networks, First Monday. 2

[CR73] Tanwar S, Parekh K, Evans R (2020). Blockchain-based electronic healthcare record system for healthcare 4.0 applications. J. Inf. Secur. Appl..

[CR74] Tanwar S, Bhatia Q, Patel P, Kumari A, Singh PK, Hong W-C (2020). Machine learning adoption in Blockchain-based smart applications: the challenges, and a way forward. IEEE ACCESS..

[CR75] Thwin TT, Vasupongayya S (2019) Blockchain-based access control model to preserve privacy for personal health record systems. Secur. Commun. Netw. 10.1155/2019/8315614

[CR76] Till BM, Peters AW, Afshar S, Meara JG (2017). From blockchain technology to global health equity: can cryptocurrencies finance universal health coverage?. BMJ Glob Health.

[CR77] Treiblmaier H (2018). The impact of the blockchain on the supply chain: a theory-based research framework and a call for action. Supply Chain Manag Int J.

[CR78] Treiblmaier H (2019). Toward more rigorous Blockchain research: recommendations for writing Blockchain case studies. Front Blockchain.

[CR79] Tseng JH, Liao YC, Chong B, Liao SW (2018) Governance on the drug supply chain via gcoin blockchain. Int. J. Environ. Res. Public. Health 15. 10.3390/ijerph15061055 10.3390/ijerph15061055 PMC6025275 29882861

[CR80] Wagner CS, Brahmakulam I, Jackson B, Wong A, Yoda T (2001) Science and Technology Collaboration: Building Capability in Developing Countries, Rand Corp Santa Monica CA,. https://apps.dtic.mil/docs/citations/ADA391917 (accessed March 29, 2020)

[CR81] Wang H, Song Y (2018). Secure cloud-based EHR system using attribute-based cryptosystem and Blockchain. J Med Syst.

[CR82] Wang S, Wang J, Wang X, Qiu T, Yuan Y, Ouyang L, Guo Y, Wang F-Y (2018). Blockchain-powered parallel healthcare systems based on the ACP approach. IEEE Trans Comput Soc Syst.

[CR83] Wang Y, Singgih M, Wang J, Rit M (2019). Making sense of blockchain technology: how will it transform supply chains?. Int J Prod Econ.

[CR84] Xia Q, Sifah EB, Asamoah KO, Gao J, Du X, Guizani M (2017). MeDShare: trust-less medical data sharing among cloud service providers via Blockchain. IEEE Access..

[CR85] Xia Q, Sifah EB, Smahi A, Amofa S, Zhang X (2017). BBDS: Blockchain-based data sharing for electronic medical Records in Cloud Environments. Information.

[CR86] Yang G, Li C, Marstein KE, A blockchain-based architecture for securing electronic health record systems, Concurr. Comput. Pract. Exp. n/a (2019) e5479. 10.1002/cpe.5479

[CR87] Yue X, Wang H, Jin D, Li M, Jiang W (2016). Healthcare data gateways: found healthcare intelligence on Blockchain with novel privacy risk control. J Med Syst.

[CR88] Zhang A, Lin X (2018) Towards Secure and Privacy-Preserving Data Sharing in e-Health Systems via Consortium Blockchain. J. Med. Syst. 42. 10.1007/s10916-018-0995-5 10.1007/s10916-018-0995-5 29956061

[CR89] Zhang J, Xue N, Huang X (2016). A secure system for pervasive social network-based healthcare. IEEE ACCESS.

[CR90] Zhang P, White J, Schmidt DC, Lenz G, Rosenbloom ST (2018). FHIRChain: applying Blockchain to securely and Scalably share clinical data. Comput Struct Biotechnol J.

[CR91] Zhang P, Schmidt DC, White J, Lenz G (2018). Blockchain technology use cases in healthcare. Blockchain Technol Platf Tools Use Cases.

[CR92] Zhou T, Li X, Zhao H (2019) Med-PPPHIS: Blockchain-Based Personal Healthcare Information System for National Physique Monitoring and Scientific Exercise Guiding. J. Med. Syst. 43. 10.1007/s10916-019-1430-2 10.1007/s10916-019-1430-2 31410583

[CR93] Zupic I, Čater T (2015). Bibliometric methods in management and organization. Organ Res Methods.

